# PCPA protects against monocrotaline-induced pulmonary arterial remodeling in rats: potential roles of connective tissue growth factor

**DOI:** 10.18632/oncotarget.22882

**Published:** 2017-12-04

**Authors:** Yang Bai, Zhong-Xia Li, Yue-Tong Zhao, Mo Liu, Yun Wang, Guo-Chao Lian, Qi Zhao, Huai-Liang Wang

**Affiliations:** ^1^ Department of Clinical Pharmacology, College of Pharmacy, China Medical University, Shenyang, 110122, China; ^2^ Department of Orthopaedic Ward, Central Hospital Affiliated Shenyang Medical College, Shenyang, 110122, China; ^3^ School of Mathematics, Liaoning University, Shenyang, 110036, China; ^4^ Research Center for Computer Simulating and Information Processing of Bio-Macromolecules of Liaoning Province, Shenyang, 110036, China; ^5^ National Key Subject, Institute of Respiratory Diseases, China Medical University, Shenyang, 110122, China; ^6^ Institute of Cardiovascular Diseases, China Medical University, Shenyang, 110122, China

**Keywords:** CTGF, PCPA, pulmonary arterial hypertension, monocrotaline, remodeling

## Abstract

The purpose of this study was to investigate the mechanism of monocrotaline (MCT)-induced pulmonary artery hypertension (PAH) and determine whether 4-chloro-DL-phenylalanine (PCPA) could inhibit pulmonary arterial remodeling associated with connective tissue growth factor (CTGF) expression and downstream signal pathway. MCT was administered to forty Sprague Dawley rats to establish the PAH model. PCPA was administered at doses of 50 and 100 mg/kg once daily for 3 weeks via intraperitoneal injection. On day 22, the pulmonary arterial pressure (PAP), right ventricle hypertrophy index (RVI) and pulmonary artery morphology were assessed and the serotonin receptor-1B (SR-1B), CTGF, p-ERK/ERK were measured by western blot or immunohistochemistry. The concentration of serotonin in plasma was checked by ELISA. Apoptosis and apoptosis-related indexes were detected by TUNEL and western blot. In the MCT-induced PAH models, the PAP, RVI, pulmonary vascular remodeling, SR-1B index, CTGF index, anti-apoptotic factors bcl-xl and bcl-2, serotonin concentration in plasma were all increased and the pro-apoptotic factor caspase-3 was reduced. PCPA significantly ameliorated pulmonary arterial remodeling induced by MCT, and this action was associated with accelerated apoptosis and down-regulation of CTGF, SR-1B and p-ERK/ERK. The present study suggests that PCPA protects against the pathogenesis of PAH by suppressing remodeling and inducing apoptosis, which are likely associated with CTGF and downstream ERK signaling pathway in rats.

## INTRODUCTION

Pulmonary artery hypertension (PAH) is a progressive and devastating disease defined by increased pulmonary arterial pressure (>25 mmHg), vascular remodeling and narrowed pulmonary arteries [1, 2]. Without treatment, PAH leads to right ventricle dysfunction, cardiac output reduced, right ventricle failure and can lead to death [3]. Smooth muscle cell proliferation, endothelial damage and remodeling are involved in the pathogenesis of PAH [4–7]. Endothelial cell injury and apoptosis are generally considered to represent PAH initiation and they are caused by several factors which can induce pulmonary arterial smooth muscle cells (PASMCs) and fibroblast proliferation as well as vascular remodeling [8–10]. Interest in the cellular and molecular mechanisms underlying the formation and development of PAH and the key role of cell apoptosis in PAH recently has seen significant increase. Apoptosis, or programmed cell death, is an automatic and ordered cell death process that is controlled by several genes which maintains homeostasis [11]. In this process, damaged cells or unwanted cells that might be disadvantageous to the organism are removed [11]. Disturbing the balance between PASMCs proliferation and apoptosis has shown to play an important role in vascular remodeling and vessel hypertrophy [12–15]. Apoptosis-resistant endothelial cells or highly proliferative PASMCs promote pulmonary vascular lesions [5]. Previous studies have shown that therapeutic strategies for PAH were limited to vasoactive agents, including prostaglandins, endothelin receptor antagonists and a phosphodiesterase type-5 inhibitor [16]. Recently, therapies based on anti-apoptotic mechanisms have been successful in animal models of pulmonary hypertension (PH) [16]. Therefore, agents designed to induce apoptosis or inhibit PASMCs growth represent effective and promising approaches.

Connective tissue growth factor (CTGF), which is also called CCN2, is a member of the CCN family of secreted peptides [17], and is derived from human umbilical vein endothelial cells (HUVECs). It is composed of four modules: insulin-like growth factor (IGF)-binding protein, thrombospondin type I, von Willebrand type C factor and COOH-terminal domain [18]. By interacting with a variety of cell surface receptors, growth factors or extracellular matrix proteins, CTGF regulates multiple cellular functions, including cell proliferation, survival, adhesion and apoptosis as well as a wide range of other biological processes [19–21]. Increasing evidences have shown the importance of anti-apoptotic effect of CTGF in disease development. The over-expression of CTGF provides a protective effect against drug-induced apoptosis via a αvβ3/FAK/extracellular signal-regulated kinase (ERK) signaling pathway, which mediates the bcl-xl survival pathway [22]. Furthermore, CTGF might be involved in the pathogenesis of PAH. Moreover, transforming growth factor-β1 (TGF-β1) and CTGF are increased in high blood flow-induced PH, and the expression of CTGF is correlated with pulmonary vascular remodeling indicators (relative medial thickness, RMT; and relative medial area, RMA) [23]. The expression of CTGF protein and mRNA were increased in monocrotaline (MCT)-treated rats [24]. However, the detailed mechanism of CTGF in the remodeling of PASMCs in the development of PAH remains unclear.

Serotonin (5-hydroxytrytamine, 5-HT) is synthesized by tryptophan hydroxylase (TPH). TPH1 is predominantly present in the peripheral nervous system, whereas TPH2 is exclusively found in the central nervous system [25]. In idiopathic PAH (IPAH), TPH1 expression and serotonin synthesis are increased in the pulmonary vascular endothelium, and the serotonin transporter (5-HTT, SERT) is increased in PASMCs [26]. 5-HT promotes the proliferation of PASMCs and pulmonary arterial fibroblasts and leads to vasoconstriction and vascular remodeling during the development of PAH [27, 28]. Reports have demonstrated that 5-HT induces vasoconstriction via serotonin receptor-1B (SR-1B) and SR-2A [29]. Researchers have found that SR-1B agonists increase proliferation and decrease apoptosis of PASMCs by pERK1/2 and PDK [30].

Our previous study showed that 4-chloro-DL-phenylalanine (PCPA) ameliorates pulmonary artery remodeling and lung tissue inflammation and decreases the expression of TPH1, matrix metalloproteinase (MMP)/tissue inhibitor of metalloproteinase (TIMP) and pro-inflammatory factors [31]. However, the effect of PCPA on apoptosis during the pathogenesis of PAH and the roles of CTGF and ERK/p-ERK as well as their relationship remain unclear. In this study, we aim to investigate the mechanism of CTGF in PAH and determine whether PCPA can inhibit pulmonary arterial remodeling by regulating CTGF and its downstream signaling pathway.

## RESULTS

### Effect of PCPA on MCT-induced PAP, RVI and pulmonary arterial wall thickness

**Figure 1:**
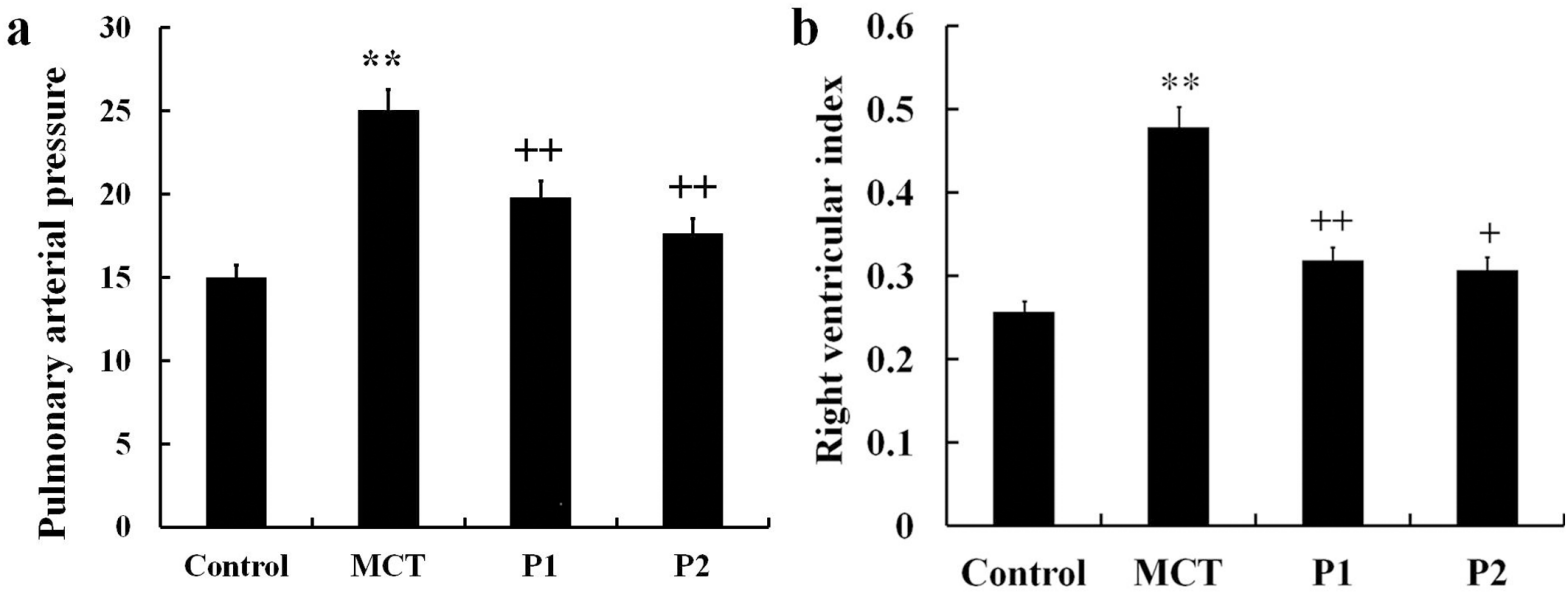
Effect of PCPA on MCT-induced PAP and RVI **(a)** Pulmonary arterial pressure (PAP) and **(b)** right ventricle hypertrophy index (RVI) were compared in different groups. Data are expressed as the means ± SD. ^**^P < 0.01 compared with the control group. ^+^ P < 0.05, ^++^ P < 0.01 compared with the MCT group. P, 4-Chloro-DL-phenylalanine (PCPA); MCT, monocrotaline; P1, MCT plus 50 mg/kg body mass PCPA; P2, MCT plus 100 mg/kg body mass PCPA.

Animals were fed for 21 days. In the MCT group, pulmonary arterial pressure (PAP) markedly exceeded to other three groups. PCPA obviously inhibited the PAP which was induced by MCT (Figure 1a). The right ventricle hypertrophy index (RVI) in the MCT group was significantly increased from 0.26 ± 0.07 to 0.48 ± 0.09 (P < 0.01 compared with the control group). Compared with the MCT group, the PCPA-treated groups showed significantly reduced RVI values, with the P1 group decreasing to 0.32 ± 0.06 (P < 0.01 *vs.* MCT) and P2 group decreasing to 0.31 ± 0.03 (P < 0.01 *vs.* MCT, Figure 1b).

**Figure 2:**
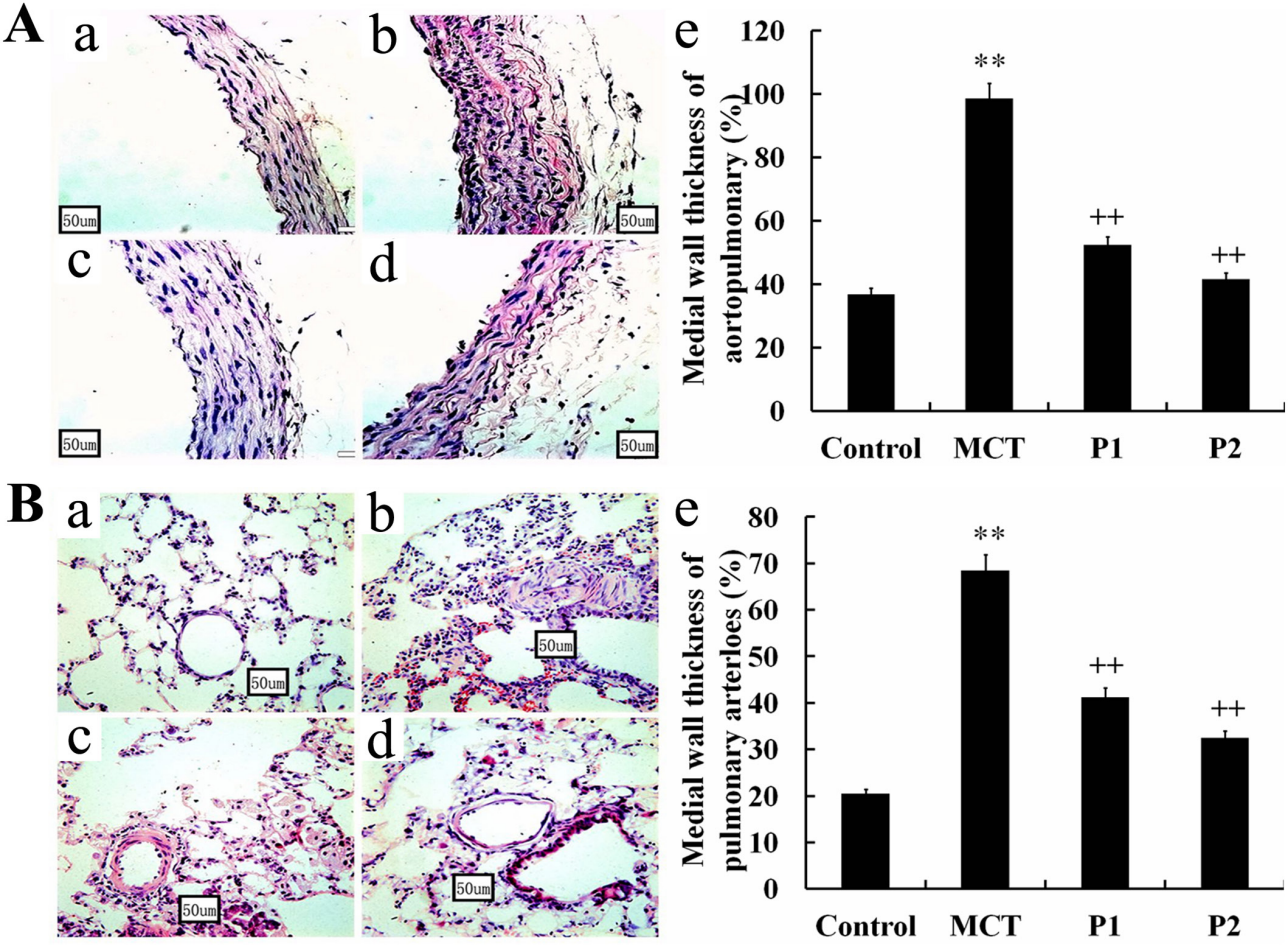
Effect of PCPA on the aortopulmonary and pulmonary arterioles wall thickness ratio Aortopulmonary **(A)** and pulmonary arterioles **(B)** obtained from **(a)** control group, **(b)** monocrotaline (MCT) group, **(c)** MCT plus 50 mg/kg body mass PCPA group (P1), and **(d)** MCT plus 100 mg/kg body mass PCPA group (P2). Pulmonary vascular remodeling measured as a percentage of **(e)** medial wall thickness in the 4 groups. (Original magnification × 400, scale bars = 50 μm). Data are the mean ± SD (n = 3 rats). ^**^ P < 0.01 compared with the control group. ^++^ P < 0.01 compared with the MCT group.

The muscularization of the pulmonary arterials and lung tissues were studied via light microscopy. PCPA dramatically suppressed the remodeling of the pulmonary arteries. In the MCT group, the thickness of the aortopulmonary wall had remarkably increased (98.51 ± 5.32%, P < 0.01 *vs.* control 36.88 ± 6.85%). PCPA suppressed the thickness ratio in the P1 and P2 groups (52.37 ± 6.87% and 41.57 ± 9.55%, respectively, P < 0.01 *vs.* MCT, Figure 2A). The pulmonary arteriole wall thickness was increased from 20.48 ± 4.69% in the control group to 68.48 ± 8.76% in the MCT group (P < 0.01), and the medial wall thickness ratio was inhibited in the P1 and P2 groups (41.24 ± 7.57% and 32.42 ± 5.69%, respectively, P < 0.01 *vs.* MCT, Figure 2B).

### Effect of PCPA on 5-HT in plasma

**Figure 3:**
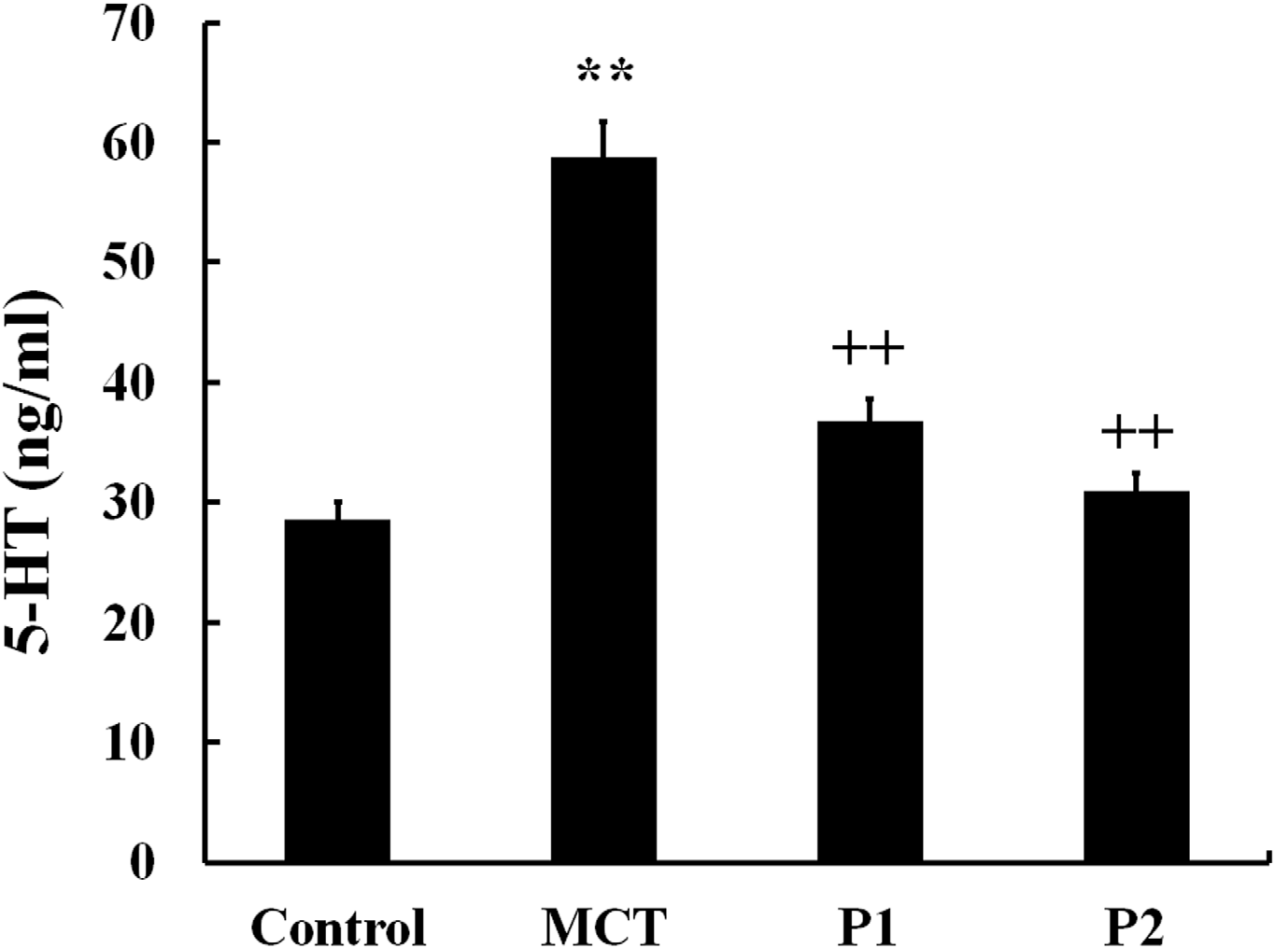
ELISA determination of PCPA on 5-HT concentration in plasma Data are expressed as the means ± SD. ^**^ P < 0.01 compared with the control group. ^++^ P < 0.01 compared with the MCT group. P, 4-Chloro-DL-phenylalanine (PCPA); MCT, monocrotaline; P1, MCT plus 50 mg/kg body mass PCPA; P2, MCT plus 100 mg/kg body mass PCPA.

To determine the role of PCPA in MCT-induced PAH, we measured 5-HT concentration in plasma in the four groups. In the control group, the 5-HT concentrations in plasma were 28.56 ± 2.5 ng/mL. In the MCT group, the 5-HT concentrations in plasma significantly increased to 58.76 ± 9.32 ng/mL (P < 0.01 vs control). At 50 mg/kg, PCPA inhibited the 5-HT concentration in plasma (36.73 ± 7.34 ng/mL, P < 0.01 vs MCT). At 100 mg/kg, PCPA markedly inhibited the plasma 5-HT concentration (30.90 ± 5.64 ng/mL, P < 0.01 vs MCT group, Figure 3).

### Effect of PCPA on CTGF expression in aortopulmonary and pulmonary arterioles

**Figure 4:**
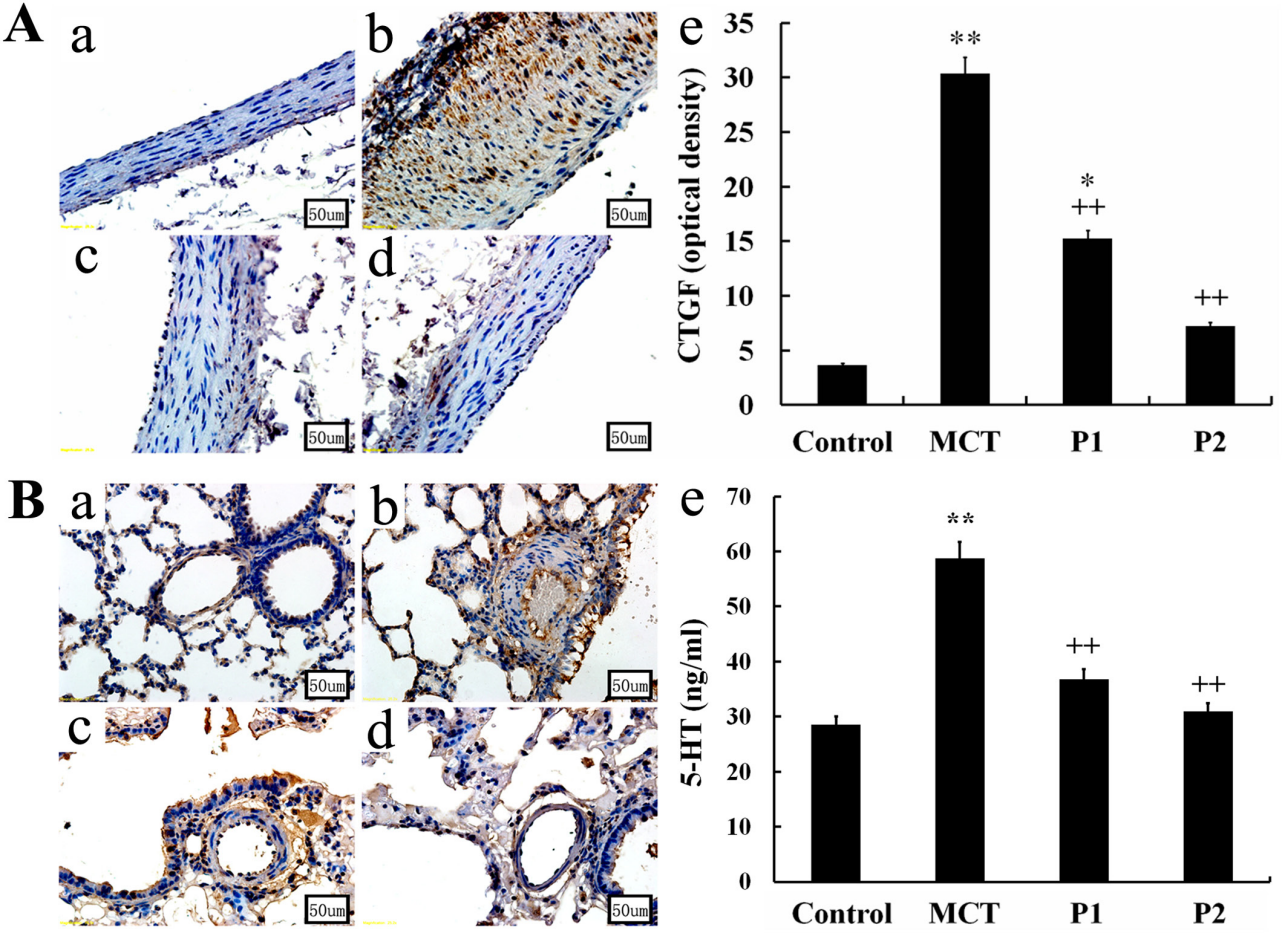
Immunohistochemical determination of CTGF expression in the aortopulmonary and pulmonary arterioles Aortopulmonary **(A)** and pulmonary arterioles **(B)** obtained from **(a)** control group, **(b)** monocrotaline (MCT) group, **(c)** MCT plus 50 mg/kg body mass 4-Chloro-DL-phenylalanine (PCPA) group (P1), and **(d)** MCT plus 100 mg/kg body mass PCPA group (P2). (Original magnification × 400, scale bars = 50 μm). **(e)** Average optical density of CTGF in rat aortopulmonary. Data are the mean ± SD (n = 3 rats). ^*^ P < 0.05, ^**^P < 0.01 compared with the control group. ^++^P < 0.01 compared with the MCT group.

Immunohistochemistry and Western blotting analyses were used to evaluate the CTGF expression in the aortopulmonary and pulmonary arterioles. The immunohistochemistry analysis showed strong positive CTGF expression in the MCT group compared with control group. The low dosage of PCPA caused a mild reduction of CTGF expression in the aortopulmonary and pulmonary arterioles, whereas the high dosage of PCPA seemingly suppressed the MCT-induced CTGF overexpression (Figure 4).

**Figure 5:**
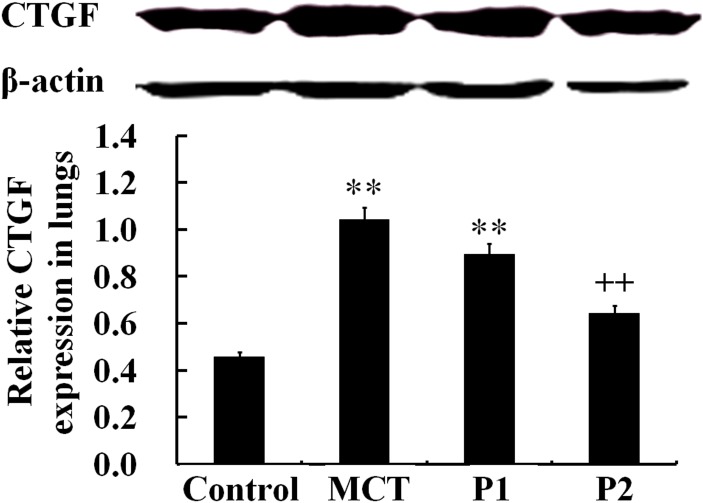
Western blot analysis of CTGF expression in lung tissues The control group, the monocrotaline (MCT)-induced pulmonary arterial hypertension group, P1 group with 4-Chloro-DL-phenylalanine (PCPA) treated 50 mg/kg/day, and P2 group with PCPA treated 100 mg/kg/day. Data are expressed as mean ± SD (n = 5 rats). ^**^ P < 0.01 compared with the control group. ^++^ P < 0.01 compared with the MCT group.

The Western blotting analysis showed that the levels of CTGF protein were strongly up-regulated in the MCT group (1.04 ± 0.08 *vs.* 0.46 ± 0.11, respectively, P < 0.01). At the 50 mg/kg dose, PCPA inhibited the MCT-induced expression of CTGF (0.89 ± 0.13, P > 0.05 *vs.* MCT). At a higher dose (100 mg/kg), PCPA markedly suppressed the MCT-induced CTGF expression (0.64 ± 0.13, P < 0.01 *vs.* MCT, Figure 5).

### Effect of PCPA on SR-1B in rat lungs

**Figure 6:**
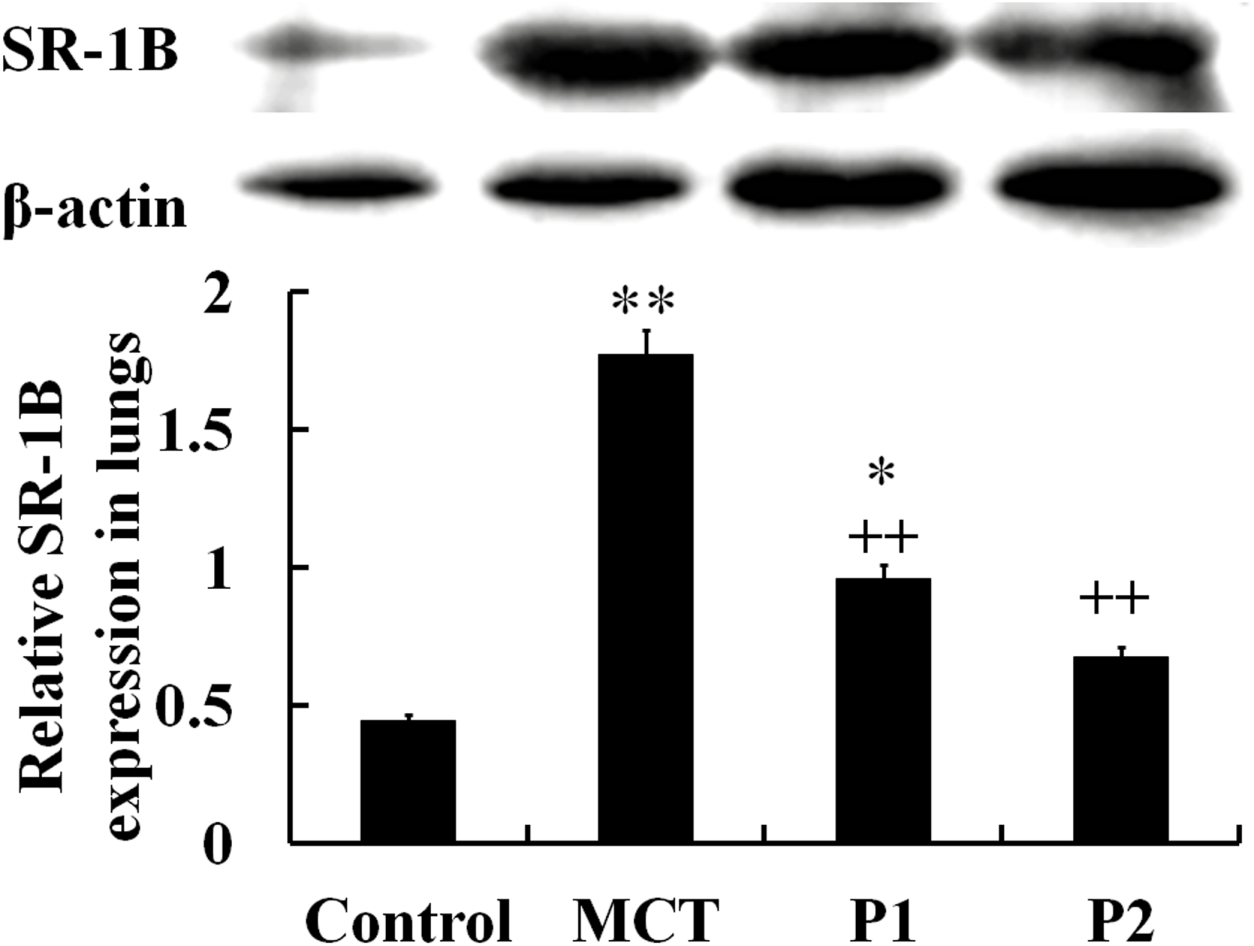
Western blot analysis of SR-1B expression in lung tissues The control group, the monocrotaline (MCT)-induced pulmonary arterial hypertension group, P1 group with 4-Chloro-DL-phenylalanine (PCPA) treated 50 mg/kg/day, and P2 group with PCPA treated 100 mg/kg/day. Data are expressed as mean ± SD (n = 5 rats). ^*^ P < 0.05, ^**^P < 0.01 compared with the control group. ^++^P < 0.01 compared with the MCT group.

The Western blotting analysis using anti-SR-1B antibodies showed that the levels of SR-1B protein significantly increased in the MCT-induced lungs compared with the control group (from 0.45 ± 0.09 to 1.77 ± 0.11, P < 0.01). Treatment with PCPA (50 mg/kg) caused a small improvement in SR-1B protein expression (0.96 ± 0.08, P < 0.01 *vs.* MCT). At 100 mg/kg, PCPA significantly attenuated the MCT-induced expression of SR-1B in lung tissues (0.68 ± 0.07, P < 0.01 *vs.* MCT, Figure 6).

### Effect of PCPA on ERK and p-ERK expression in rat lungs

**Figure 7:**
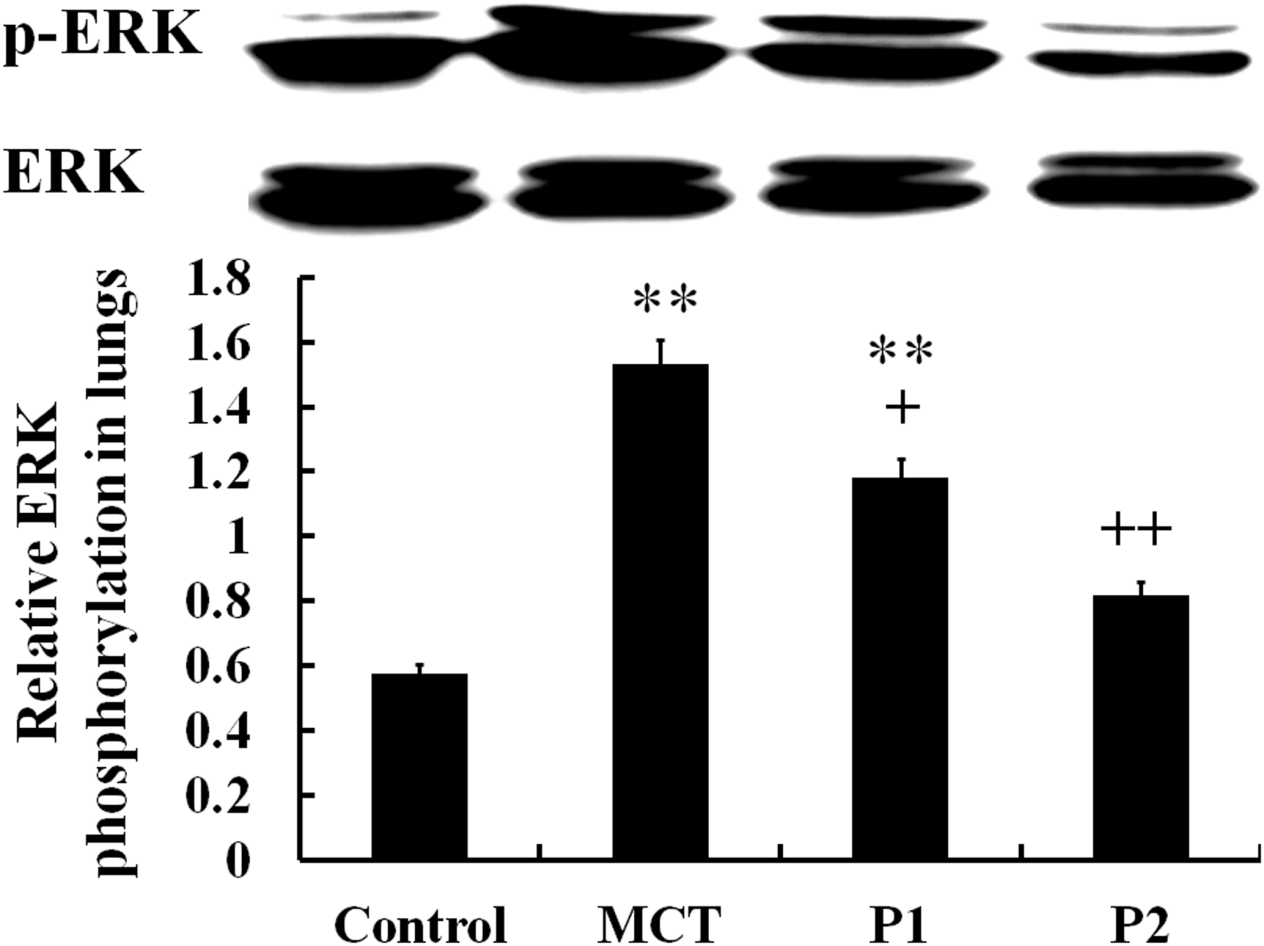
Comparison of ERK phosphorylation in rat lung tissues The control group, the monocrotaline (MCT)-induced pulmonary arterial hypertension group, P1 group with 4-Chloro-DL-phenylalanine (PCPA) treated 50 mg/kg/day, and P2 group with PCPA treated 100 mg/kg/day. Data are expressed as mean ± SD (n = 5 rats). ^**^ P < 0.01 compared with the control group. ^+^ P < 0.05, ^++^P < 0.01 compared with the MCT group.

The Western blotting analysis of ERK phosphorylation demonstrated that MCT-induced ERK phosphorylation was significantly up-regulated compared with that of the control group from 0.58 ± 0.05 to 1.53 ± 0.13 (P < 0.01). PCPA inhibited the induction of ERK phosphorylation by MCT. The levels of p-ERK/ERK decreased to 1.18 ± 0.05 (P < 0.05 *vs.* MCT) in the P1 group and 0.82 ± 0.07 (P < 0.01 *vs.* MCT) in the P2 group. Together, these results show that PCPA inhibited the MCT-induced activation of ERK in rat lungs (Figure 7).

### Effect of PCPA on apoptosis in rat lungs

**Figure 8:**
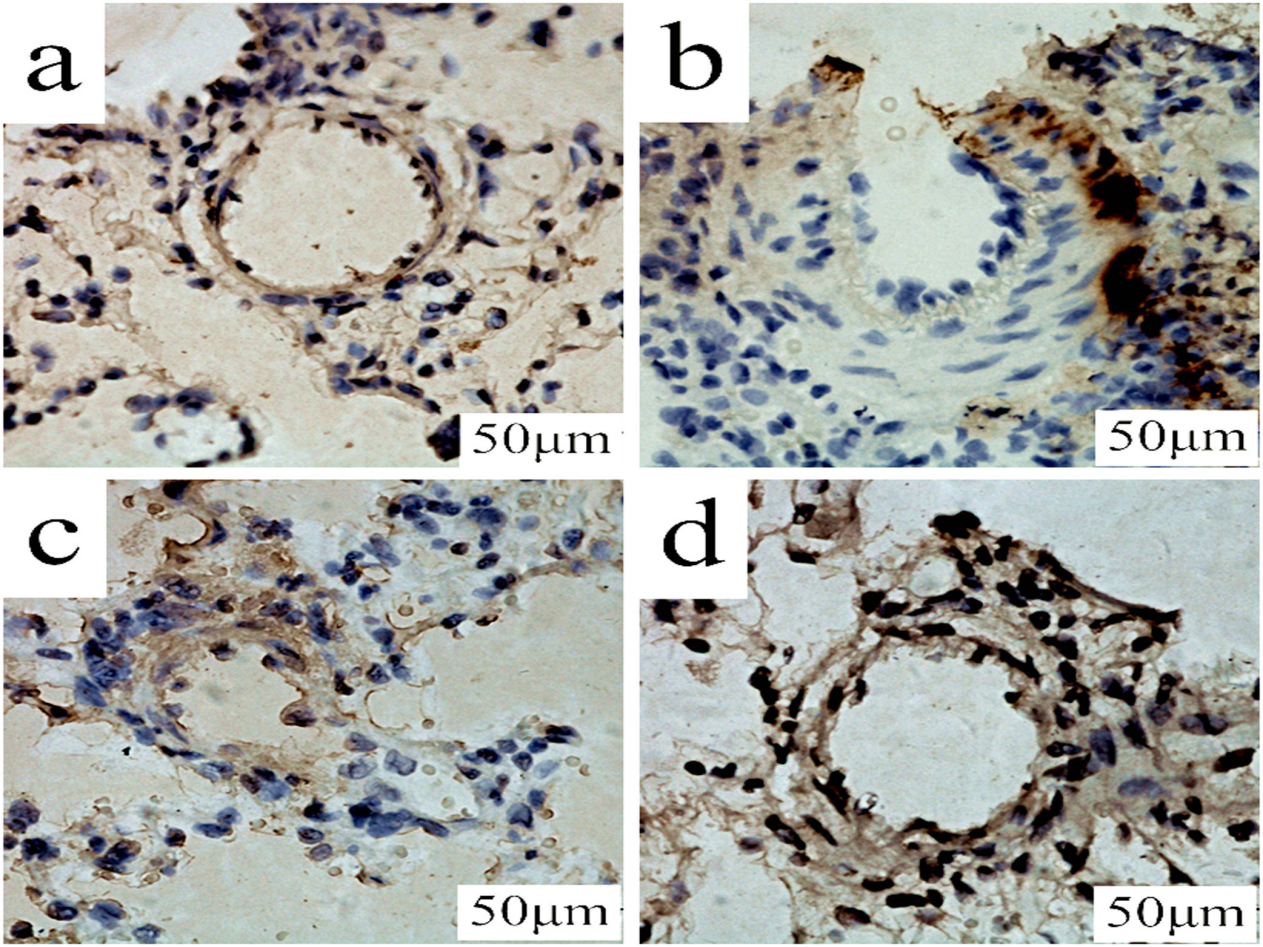
TUNEL determination of PCPA on apoptosis in lung tissues Lung tissues obtained from **(a)** control group, **(b)** monocrotaline (MCT) group, **(c)** MCT plus 50 mg/kg body mass 4-Chloro-DL-phenylalanine (PCPA) group (P1), and **(d)** MCT plus 100 mg/kg body mass PCPA group (P2). (Original magnification × 400, scale bars = 50 μm).

As shown in Figure 8, in MCT group, the percentage of TUNEL-positive cells were slightly decreased (vs control group). PCPA treatment evidently enhanced the percentage of TUNEL-positive cells, especially with high dosage.

**Figure 9:**
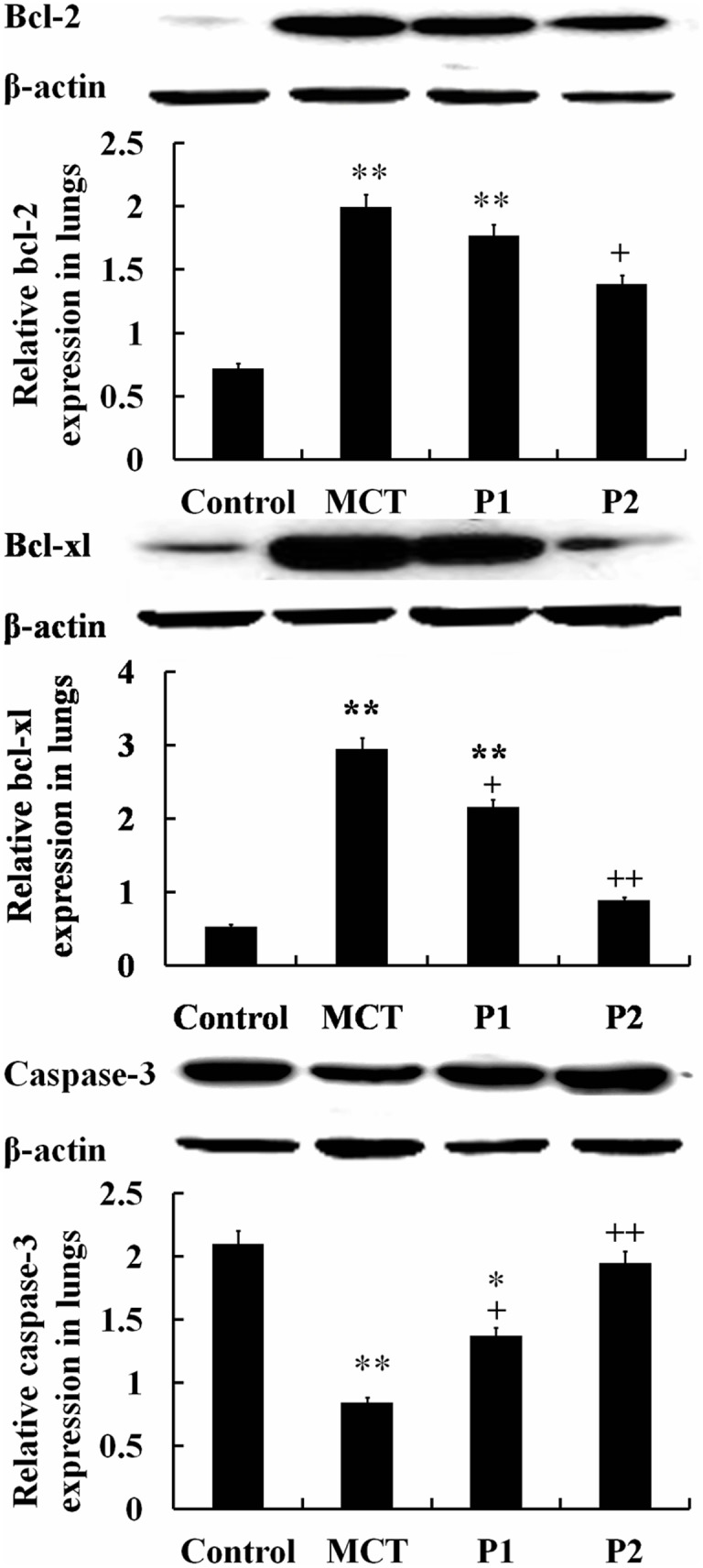
Western blot analysis of bcl-2, bcl-xl and caspase-3 protein expression in lungs The control group, the monocrotaline (MCT)-induced pulmonary arterial hypertension group and the 50 and 100 mg/kg 4-Chloro-DL-phenylalanine (PCPA) treated groups (P1 and P2, respectively). Data are expressed as mean ± SD (n = 5 rats). ^**^P < 0.01 compared with the control group. ^+^P < 0.05, ^++^P < 0.01 compared with the MCT group.

The Western blotting analysis showed that the levels of bcl-2 and bcl-xl in the MCT group were noticeably up-regulated compared with that of the control group. In the MCT group, bcl-2 expression increased from 0.72 ± 0.39 to 1.99 ± 0.24 (P < 0.01 *vs*. control) and bcl-xl increased from 0.53 ± 0.09 to 2.95 ± 0.05 (P < 0.01 *vs*. control). PCPA inhibited bcl-2 and bcl-xl expression. In the P1 group, bcl-2 and bcl-xl protein decreased to 1.77 ± 0.06 and 2.16 ± 0.07 (P < 0.05 *vs*. MCT, respectively). In the P1 group, bcl-2 and bcl-xl protein were reduced to 1.39 ± 0.21 (P < 0.05 *vs*. MCT) and 0.89 ± 0.30 (P < 0.01 *vs*. MCT), respectively. However, the levels of capase-3 significantly decreased in the MCT group from 2.09 ± 0.41 in the control group to 0.84 ± 0.16 (P < 0.01). PCPA up regulated the expression of capase-3, especially at the higher dose. In the P1 and P2 groups, capase-3 expression increased to 1.37 ± 0.13 (P < 0.05 *vs*. MCT) and 1.94 ± 0.06 (P < 0.01 *vs*. MCT), respectively (Figure 9).

## DISCUSSION

In this study, we showed that PCPA can ameliorate MCT-induced PAH which might associate with down-regulated SR-1B, CTGF and its downstream signaling pathway. Our results indicated that the expression of 5-HT, SR-1B, CTGF, p-ERK/ERK, bcl-2 and bcl-xl is significantly increased and the expression of caspase-3 is decreased in the MCT group. PCPA inhibits pulmonary artery remodeling, attenuated the expression of 5-HT, SR-1B, CTGF, p-ERK/ERK, bcl-2 and bcl-xl and increasing the expression of caspase-3. These results suggest PCPA contributes to preventing the pathogenesis of PAH by suppressing remodeling and inducing apoptosis, and these effects are likely associated with the CTGF and ERK signaling pathway.

Studies have found that disturbing the balance between proliferation and apoptosis of PASMCs is important for vascular remodeling [12–15]. MCT is a toxic alkaloid that can induce PAH via endothelial damage and PASMCs proliferation and hypertrophy. Previous results from our laboratory showed that an i.p. injection of MCT induces vascular remodeling, increases PASMC proliferation and decreases apoptosis by up-regulating anti-apoptotic proteins [31, 32]. PCPA, as an inhibitor of 5-HT synthesis, has amelioration effect on remodeling in the PAH [31, 33]. But whether PCPA can regulate the apoptosis and its associated signaling pathway is unknown. Therefore, we aim to study the involvement of CTGF and p-ERK/ERK signaling in the pathogenesis of PAH for further investigation.

Serotonin plays an important role in PAH. Results from our laboratories showed that the rate-limiting enzyme of serotonin synthesis, TPH1 and SERT expression is increased in PAH [31, 33]. Serotonin mediates PAH by inducing pulmonary vascular remodeling and vasoconstriction [27]. 5-HT leads to vascular remodeling by stimulating PASMCs proliferation and reducing apoptosis through SR or SERT [30]. The 5-HT signaling pathway is an important mechanism of human PASMCs proliferation in which SR-1B mediates MEK/ERK activation and increases the expression of many transcription factors, which then induce calcium-dependent PASMC proliferation [34]. Moreover, agents targeting 5-HT receptors or SERT may alter apoptotic pathways and ameliorate the development of vascular remodeling [34]. In addition, researchers have used SB2116641, an antagonist of SR-1B, to study cellular functions and found that it could induce apoptosis by decreasing caspase-3, -8 and -9 and poly ADP-ribose polymerase (PARP) [35]. Furthermore, 5-HT promotes vasoconstriction via SR-1B in human pulmonary arteries [36], and knockdown of the SR-1B gene and treatment with neutralizing antibodies promote effective results in a hypoxia-induced PAH animal model [36]. The data from other studies are consistent with those of our study in which the expression of TPH-1 and SR-1B were up-regulated in MCT-induced PAH and TPH-1, SR-1B and MCT-induced pulmonary arterial remodeling were decreased by PCPA. However, the regulatory mechanisms underlying these effects are not clear.

CTGF regulates a variety of cellular functions, including adhesion, proliferation, differentiation, extracellular matrix (ECM) remodeling as well as apoptosis by interacting with multiple integrins [19–21]. Increasing evidences have shown the important role of CTGF in apoptosis during disease development. The up-regulation of CTGF reduced chemotherapy-induced apoptosis in breast cancer by increasing the expression of bcl-xl and cellular inhibitor of apoptosis proteins (cIAP1) [22]. Silencing CTGF expression suppresses the growth and differentiation of rhabdomyosarcoma cells by inducing apoptosis [37]. In human chondrosarcoma, micro-environmental stress, such as hypoxia, induce the expression of CTGF via interactions between the 3’-untranslated region and a cellular protein [38]. Conversely, CTGF protects tumor cell growth by suppressing hypoxia-induced apoptosis [39]. Knockdown of CTGF in fibroblast-like synoviocytes of rheumatoid arthritis induce apoptosis and inhibits survivin mRNA expression [40]. These results indicate that CTGF is an important survival (or apoptosis) factor and could be a therapeutic target in various diseases. The important role of CTGF in the pathogenesis of PAH can be observed from many aspects. For example, Zhu et al. [23] performed a dynamic evaluation and found that the expression of TGF-β1 and CTGF was correlated with the severity of PAH and indicated that CTGF might be involved with vascular remodeling in high blood flow-induced PH. Another report indicated that the expression of CTGF protein and mRNA was increased in MCT-treated rats compared with the control groups, and this effect could be reversed by simvastatin [24]. These findings are consistent with our results. The expression of CTGF and apoptosis-associated factors was remarkably elevated in the MCT group. PCPA inhibited the expression of CTGF, bcl-2, and bcl-xl and up-regulated the expression of caspase-3 protein. Therefore, we infer that CTGF may participate in pulmonary arteries remodeling and apoptosis in MCT-induced PAH and PCPA may inhibit pulmonary artery remodeling and induce apoptosis through suppressing the TPH-1, SR-1B, which influence CTGF and the downstream signaling pathways. However, the specific mechanisms underlying these changes require further investigation.

ERKs are members of the MAPK family and widely expressed protein kinases that are involved in multiple functions, including growth and differentiation [41]. Previous studies showed that the role of ERK1/2 as an important protein kinase intracellular signaling molecules was critical in the resistance to apoptosis in various types of cells [42]. Additionally, an ERK-activating kinase inhibitor was used to block the ERK activation and reverse its protective effect against DNA strand damage and apoptosis in hyperoxic lung injury, thus indicating that this pathway is ERK dependent [43]. Furthermore, studies have found the Ras-MAPK pathway promotes cell survival and inhibits apoptosis by increasing the transcription of pro-survival genes and suppressing various apoptotic regulatory molecules, including Bad, Bim, Mcl-1, caspase-9 and bcl-2, via phosphorylation [41, 44]. In dexamethasone-induced thymocyte cells apoptosis, ERK1/2 is suppressed, which causes calcium ion pool to dysfunction and prevents the phosphorylation of bcl-2, thereby accelerating the apoptotic process [45, 46]. Moreover, ERK/MAPK had been demonstrated to be involved in MCT-induced PAH and aspirin treatment attenuates PAH by suppressing ERK1/2 signaling pathway in rats [47]. Our present study showed that in MCT-induced PAH the expression of p-ERK/ERK and apoptosis-resistant associated proteins such as bcl-2, bcl-xl were up-regulated, but pro-apoptosis protein caspase-3 was inhibited. PCPA inhibited the p-ERK/ERK signal pathway and induced the apoptosis, which indicated that the ERK signaling pathway may regulate the process of apoptotic in the MCT-induced PAH and therefore ameliorate the pulmonary arteries remodeling.

Additionally, previous research had shown that the COOH-terminal domain of CTGF binds to integrin αvβ3 which activates FAK and ERK1/2 and increases the expression of bcl-xl in MCF7 cells [22]. Αvβ3 integrin is necessary for CTGF-induced ERK activation [22]. Evidence had also shown that anti-apoptotic effect of CCR7 is related to the downstream bcl-2, bax and caspase-3; however, treatment with PD98059 indirectly inhibited ERK activation via the MEK pathway and reverses the CCR7 effect [48]. During cisplatin treatment of human osteosarcoma, CTGF could protect against apoptosis by enhancing the expression of bcl-xl and survivin [49]. Further investigations had shown that CTGF exerted anti-apoptotic effects via the FAK, MEK, and ERK signaling pathways [49]. A recent study showed that the up-regulation of CTGF significantly reduced its apoptotic effect in 5-FU-treated CRC cells [50]. Factors upstream of CTGF can enhance the phosphorylation of ERK signaling pathways as well as the expression of bcl-xl and survivin [50]. These results indicated that CTGF can regulate the apoptosis by activating the phosphorylation of ERK, and based on our experimental results, we conclude that PCPA may suppress the expression of CTGF via the p-ERK/ERK signal pathway and then promote apoptosis and inhibit pulmonary artery remodeling. Thus, by targeting defects in anti-apoptosis signaling pathways, PCPA might exert apoptotic effects in PAH rats.

Accumulating experimental studies and computational models have shown that non-coding RNAs (ncRNAs) are involved in the life cycle of cells through different mechanism and play an important role in various biological processes [51–56]. NcRNAs can be divided into long ncRNAs (lncRNA) and small ncRNA (including siRNA, miRNA and piRNA, et al) [52]. Considering the different functions of ncRNAs, it is no hard to find that the dysregulations and mutations of ncRNAs are tightly associated to the progression of remodeling in PAH [57–59]. Although the roles of ncRNAs in PAH has been some progress, but the specific mechanisms need to be further studied.

In conclusion, the increased proliferation and decreased apoptosis of pulmonary arteries plays an important role in PASMC accumulation and pulmonary blood vessel remodeling. Considerable evidence support the effect of 5-HT on pulmonary vasculature in the pathobiology of PAH. PCPA acts as an inhibitor of TPH1 to reduce the levels of TPH and synthesis of 5-HT. Our results indicated that decreased levels of TPH1 suppresses pulmonary arterial remodeling and induces apoptosis which is most likely mediated by SR-1B, and this may involve CTGF and p-ERK/ERK signaling. Extensive research is necessary in order to investigate the specific mechanism underlying these effects, their relationship, and uncovering further potential therapeutic targets to treat PAH.

## MATERIALS AND METHODS

### Animal models

Forty Sprague-Dawley (SD) rats weighing 180 ± 10 g were obtained from the Animal Resource Center in China Medical University (Certificate No. Liaoning 034). All rats were randomly divided into four groups: the control group, in which rats received a vehicle (0.9% physiological saline); the MCT group (Sigma-Aldrich, St Louis, MO, USA) group, in which rats received MCT (60 mg/kg); the P1 group, in which rats received MCT (60 mg/kg) once and PCPA (50 mg/kg) daily; and the P2 group, in which rats received MCT (60 mg/kg) once and PCPA (100 mg/kg) daily. All rats in the MCT, P1 and P2 groups received intraperitoneal (i.p.) injections of MCT dissolved in vehicle (1:4 mixture of dehydrated ethanol-normal saline]. Rats in the PCPA-treated groups were administered PCPA (i.p.) once daily for 21 days. The control group rats received the same volume of vehicle as the other groups during the same period. All rats were provided food and water *ad libitum* and placed under a natural day/night cycle at 18-22°C and 50-70% humidity. All measurements taken throughout the entire experiment were performed in a blinded fashion. All animal experiments were conducted in strict accordance with international ethical guidelines and the National Institute of Health Guide concerning the Care and Use of Laboratory Animals. The protocol was approved by the Institutional Animal Care and Use Committee (IACUC) of China Medical University. The approval reference number is SYXK (Liao) 2013-0007. All research was accepted by the local authority and conducted according to the guidelines of China Medical University.

### Detection of pulmonary arterial morphometry and evaluation of right ventricular hypertrophy

On day 22, the rats were anaesthetized via the administration of 3% pentobarbital sodium (40 mg/kg), and then the PAP and systemic arterial pressure were measured using methods detailed in our previous study [31]. Then, the rats were euthanized with pentobarbital sodium (60 mg/kg, absolute lethal dose, i.p.), and heart and lung tissues were excised. The hearts were separated into the left ventricle plus septum (LV + S) and the right ventricle (RV). The RVI was calculated using the formula RVI = RV/(LV + S) to evaluate heart remodeling.

We used 4% paraformaldehyde and sterile saline to exsanguinate the rats. The pulmonary arteries and lungs tissues were cut and placed in 4% paraform. Paraffin-embedded pulmonary arteries and lung tissues were prepared in 5-μm sections and then stained with hematoxylin-eosin (H&E) for further research. For each rat, 15 pulmonary arteries were observed using a MetaMorph (Universal Imaging Corporation, PA, USA)-DP10/BX51 system (Olympus, Tokyo, Japan). We used the following formula to calculate the medial thickness for measuring pulmonary arterial remodeling: pulmonary arterial wall thickness (%) = (external diameter – internal diameter)/external diameter × 100% [31].

### Immunohistochemical staining

Paraffin-embedded aortopulmonary and lung tissues sections were dyed using ultrasensitive S-P and diaminobenzidine (DAB) staining kits (Maxin-Bio, Fuzhou, China). The primary rabbit polyclonal antibody against CTGF (BA0752, Boster biotechnology) was diluted to 1:200. The negative control was incubated in 0.01 M phosphate-buffered saline (PBS) instead of the primary antibody. A BX51 microscope (Olympus) was used to analyze the digital pictures. Fifteen pulmonary arteries (external diameter 50∼100 μm) from at least 3 rats were analyzed in each group, and at least 3 aortopulmonary sections were studied among the 4 groups. The levels of CTGF were assessed as the average optical density. Immunohistochemical staining followed a basic indirect protocol using a citrate antigen-retrieval method [31].

### ELISA analysis

We collected blood samples from different groups of rats in the chilled tubes containing 2 mg/ml EDTA. Then the tubes were centrifuged at 2400 g for 10 min (4°C). The plasma was collected frozen and stored at −80°C for ELISA. The concentrations of 5-HT in plasma were measured using a rat 5-HT ELISA kit (CSB-E08364r, Cusabio, Wuhan, China) according to the manufacturer’s instructions. The limit of detection of the assay for 5-HT protein was 0.02 ng/ml.

### TUNEL

Apoptotic were determined by the terminal deoxyribonucleotidyl transferase-mediated dUTP–digoxigenin nick end-labelling (TUNEL) method of an *in situ* cell death detection kit (KGA702, Keygenbio, Nanjing, China) in terms of manufacturer’s instruction. The number of TUNEL-positive cells in 12 fields for each section of small-pulmonary arteries was quantitatively estimated as a percentage of total smooth muscle cells (×400) by researcher blinded to the treatment group.

### Total protein preparation

Total protein was prepared as previously described [31]. The lung samples were homogenized to extract the total protein content using a polytron homogenizer (Kinematica, Lucerne, Switzerland). Then, we centrifuged the homogenate at 15000 g for 30 min at 4°C. The supernatant was collected and stored at −80°C until it was used in the experiments. The total protein concentration was determined by the BCA method (Beyotime Biotechnology, Inc).

### Western blotting analysis

Equal amounts of protein were separated by a reducing SDS-PAGE and electrotransferred onto a polyvinylidene difluoride (PVDF) membrane [31, 32]. After incubation in blocking buffer (5% nonfat dry milk, TBS and 0.05% Tween-20) at room temperature for 2 h, the membranes were incubated with primary antibodies overnight at 4°C. The antibodies included rabbit polyclonal anti-CTGF antibody (1:400, BA0752, Boster Biotechnology), rabbit polyclonal anti-SR-1B antibody (1:500, BS2765, Bioworld Technology, Inc.), rabbit polyclonal anti-ERK1/2 antibody (1:500, sc-292838, Santa Cruz Biotechnology), mouse monoclonal anti-p-ERK1/2 (1:500, sc-81492, Santa Cruz Biotechnology), rabbit polyclonal anti-bcl-2 antibody (1:500, BS1511, Bioworld Technology, Inc.), rabbit polyclonal anti-bcl-xl antibody (1:500, BS1032, Bioworld Technology, Inc.), rabbit polyclonal anti-caspase-3 antibody (1:400, BS61583, Bioworld Technology, Inc.) and mouse polyclonal anti-β-actin antibody (1:2000, sc-47778, Santa Cruz Biotechnology]. The immunoreactive bands were visualized using the corresponding horseradish peroxidase-conjugated secondary antibodies and super ECL plus (Thermo Fisher Scientific, Waltham, MA, USA). The relative protein expression was quantified by densitometry using Quantity One (Bio-Rad Laboratories, California, USA).

### Statistical analysis

Data are shown as the mean with the standard error. All statistical analyses were performed using SPSS, version 16.0 (SPSS, Inc, Chicago, IL, USA). Statistical comparisons were conducted using a one-way ANOVA and Fisher’s least significant difference (LSD) or Dunnett’s T3 tests. Values of P < 0.05 were considered statistically significant.
